# Hybrid Cathode Interlayer Enables 17.4% Efficiency Binary Organic Solar Cells

**DOI:** 10.1002/advs.202105575

**Published:** 2022-01-18

**Authors:** Hang Song, Dingqin Hu, Jie Lv, Shirong Lu, Chen Haiyan, Zhipeng Kan

**Affiliations:** ^1^ Chongqing Institute of Green and Intelligent Technology Chinese Academy of Sciences Chongqing 400714 China; ^2^ College of Materials Science and Engineering Chongqing University of Technology Chongqing 400054 China; ^3^ Chongqing University 174 Shazhengjie, Shapingba Chongqing 400044 China; ^4^ Chongqing School University of Chinese Academy of Sciences (UCAS Chongqing) Chongqing 400714 China

**Keywords:** cathode interlayer, charge transfer, hybrid interface, organic solar cells

## Abstract

With the emergence of fused ring electron acceptors, the power conversion efficiency of organic solar cells reached 19%. In comparison with the electron donor and acceptor materials progress, the development of cathode interlayers lags. As a result, charge extraction barriers, interfacial trap states, and significant transport resistance may be induced due to the unfavorable cathode interlayer, limiting the device performances. Herein, a hybrid cathode interlayer composed of PNDIT‐F3N and PDIN is adopted to investigate the interaction between the photoexcited acceptor and cathode interlayer. The state of art acceptor Y6 is chosen and blended with PM6 as the active layer. The device with hybrid interlayer, PNDIT‐F3N:PDIN (0.6:0.4, in wt%), attains a power conversion efficiency of 17.4%, outperforming devices with other cathode interlayer such as NDI‐M, PDINO, and Phen‐DPO. It is resulted from enhanced exciton dissociation, reduced trap‐assisted recombination, and smaller transfer resistance. Therefore, the hybrid interlayer strategy is demonstrated as an efficient approach to improve device performance, shedding light on the selection and engineering of cathode interlayers for pairing the increasing number of fused ring electron acceptors.

## Graphical abstract

Hybrid cathode interlayer composed of PNDIT‐F3N:PDIN (0.6:0.4, in wt%) is applied in organic solar cells with PM6:Y6 as the active layer, and a power conversion efficiency of 17.4% is attained, outperforming devices with interlayers such as NDI‐M, PDINO, and Phen‐DPO. The hybrid strategy is demonstrated as an efficient approach to improve the performance of organic solar cells with nonfullerene acceptors.

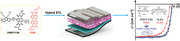

## 1 Introduction

Organic solar cells (OSCs) have emerged as a promising application prospect in flexible and portable devices owing to their unique superiorities such as low cost, lightweight, and large area printable fabrications.^[^ ^1^, ^2^, ^3^, ^4^, ^5^, ^6^ ^]^ Benefitting from the rapid development of the electron donor and acceptor materials, the power conversion efficiencies (PCEs) of single junction OSCs has been approaching 19%.^[^ ^7^, ^8^, ^9^ ^]^ It is worth noting that the PCEs cannot retain when upscaling the device area from laboratory size to large area modules. Thus, further improvement in the performance of the OSCs is of significance to facilitate the industrialization of organic photovoltaics. Synthesis of novel electron donor and acceptor materials is the primary approach to improve the PCEs. Especially, with the emergence of fused ring electron acceptors (FREAs), the performance of OSCs composed of PM6 and Y6 broke the threshold of 15%.^[^ ^10^ ^]^ Apart from the design and synthesis of novel materials, alternative strategies are evidenced as efficient methods to enhance the PCEs as well: 1) optimization of the donor/acceptor networks in the active layer,^[^ ^11^, ^12^, ^13^, ^14^ ^]^ 2) configuration of device architecture,^[^ ^15^, ^16^, ^17^, ^18^, ^19^ ^]^ and 3) engineering of cathode and/or anode interlayers.^[^ ^20^, ^21^, ^22^, ^23^ ^]^ For instance, to optimize the donor and acceptor network, processing additives were widely used to get better performance. Lv and co‐authors reported when proper solvent additives were added to prepare solutions before spin‐coating the active layer, the miscibility of the donor and acceptor can be tuned.^[^ ^24^ ^]^ As consequence, the acceptor contribution was significantly improved due to the variations in solubility and boiling point of the solvent additives. Pseudo bilayer organic solar cells were recently reported, and with this layer‐by‐layer processing method gradient donor and acceptor distribution in the active layer were formed, leading to PCE as high as 18.2% with this device architecture.^[^ ^25^ ^]^ By management of the light absorption in front and rear cells, Wang and coauthors reported tandem organic solar cells with 19.6% efficiency.^[^ ^26^ ^]^ In comparison, engineering of interface layers in OSCs is simple yet effective approach to enhance the PCEs. Poly(3,4‐ethylenedioxythiophene) polystyrene sulfonate (PEDOT:PSS) is one of the commonly used anode interlayers, which can match the highest occupied molecular orbitals (HOMO) of various electron donor materials. To inject or collect electrons from the lowest unoccupied molecular orbitals (LUMO) of acceptor, materials such as low work function metals, metal oxides, organic small molecules, and polyelectrolytes were used as cathode interlayer.^[^ ^27^, ^28^, ^29^ ^]^ Though modifications of PEDOT:PSS were reported to tune the work function and surface energy of the substrate,^[^ ^30^, ^31^, ^32^, ^33^, ^34^ ^]^ more attentions were attracted to develop the cathode interlayer materials and engineering.

With the progress of FERAs, low work function metals (e.g., calcium) and metal oxides (e.g., zinc oxide nanoparticles) were rarely used in the conventional devices. On the contrary, organic small molecules and polyelectrolytes were dominating cathode interlayers.^[^ ^22^, ^35^, ^36^ ^]^ Considering the excellent intrinsic electron transport property of perylene diimides and naphthalene diimides, cathode interlayer as PDIN, PDINO, PNDIT‐F3N, and NDI‐B were reported.^[^ ^35^, ^36^, ^37^, ^38^, ^39^ ^]^ Zhang and coauthors presented that when they replaced the cathode interlayer calcium with PDIN or PDINO, the high conductivities, appropriate energy levels and work function tuning effects make the OSCs performing well with a wide range of interlayer thickness. Notably, Jiang and coauthors used PNDIT‐F3N as the cathode interlayer when they fabricated devices with PM6:N3 as the active layer, and efficiency of 16.74% was obtained, which was the best PCE when reported.^[^ ^40^ ^]^ Besides small molecules, polyelectrolyte are key cathode interlayers as well. For example, conjugated polyelectrolyte PFN was early used as the cathode interlayer and efficiency over 9% was achieved with active layer composed of PTB7:PC_71_BM.^[^ ^41^ ^]^ The interfacial dipole formed with positive end pointing to the active layer, lowering the work function of ITO to 4.1 eV.^[^ ^42^ ^]^ Similarly, Zhou and coauthors found that nonconjugated polyelectrolyte PEI and PEIE with neutral amine groups can lower the work function when spin coated on various substrates.^[^ ^43^ ^]^ To categorize cathode interlayer by functions, though the chemical structures of abovementioned materials are varied, in common these cathode interlayers can induce interfacial dipole to shift the vacuum level, forming barrierless contact between the metal cathode and the photoactive layer and minimizing the energy loss in charge extraction.^[^ ^44^, ^45^ ^]^ However, interactions such as charge transfer from the photoexcited acceptor to the mentioned cathode interlayers were seldom discussed in organic solar cells with FERAs.

Herein, hybrid cathode interlayer composed of PNDIT‐F3N and PDIN was adopted to investigate the interaction between the photoexcited acceptor and cathode interlayer. We fabricated devices with the state of art materials PM6:Y6 as the photoactive layer. The best performance was yielded when the PNDIT‐F3N:PDIN ratio is 0.6:0.4 (in wt%). Charge transfer from the acceptor Y6 to the hybrid interlayer was confirmed by the increased photoluminescence quenching and the improved performance of devices with Y6 alone as the active layer. As a result, the device with the hybrid interlayer showed enhanced exciton dissociation, reduced trap‐assistant recombination, and smaller transport resistance in comparison with those of devices contain only one cathode interlayer, i.e., PNDIT‐F3N and PDIN based devices. Finally, the device with hybrid interlayer PNDIT‐F3N:PDIN (0.6:0.4) shows a *J*_SC_ of 27.12 mA cm^−2^, and a FF of 74.45%, leading to an elevated PCE of 17.4%. Our results demonstrate that the hybrid interlayer induced charge transfer provides a promising way to enhance the photovoltaic performance of OSCs.

## 2 Results and Discussions

**Figure 1:**
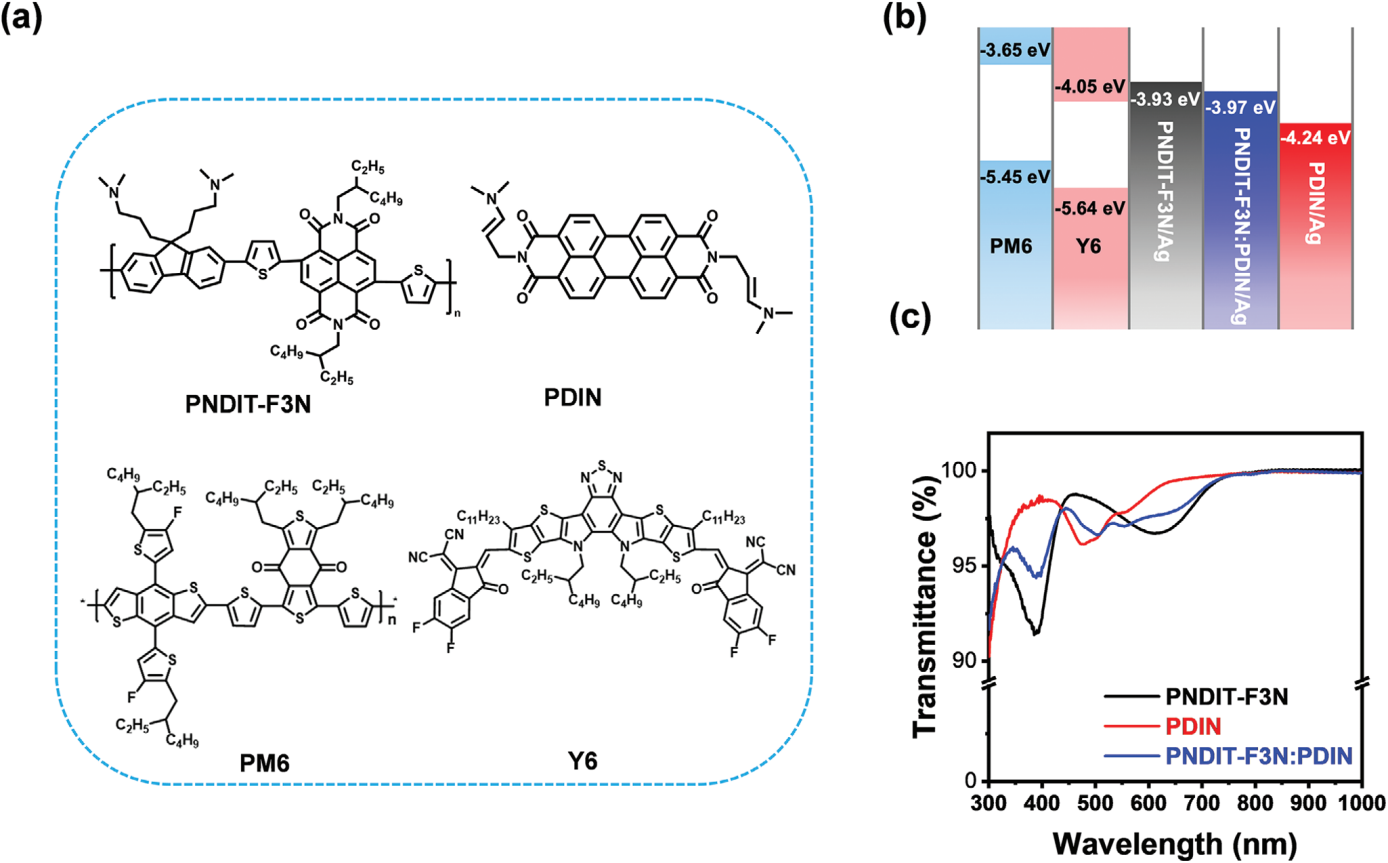
a) Chemical structures of the cathode interlayer and active layer materials. b) Energetic levels of each functional layer. c) Transmittance spectra of PNDIT, PDIN, and PNDIT‐F3N:PDIN (0.6:0.4 in wt%).

The chemical structures of the material used in this contribution are presented in **Figure** **1**a, where PNDIT‐F3N and PDIN are the selected cathode interlayers, and PM6 and Y6 are the electron donor and acceptor. The energy levels of the cathode interlayer treated electrode were determined by UPS, while the HOMO/LUMO levels of PM6/Y6 were measured with cyclic voltammetry.^[^ ^46^, ^47^ ^]^ With the treatment of PDIN, PNDIT‐F3N, and the hybrid interlayer, the work function of the silver electrode changed from −4.4 to −4.24, −3.93, and −3.97 eV, respectively. The hybrid interlayer was composed of PNDIT‐F3N and PDIN with ratio in weight (0.6:0.4), which was determined from the optimization process of OSCs in later sections. The transmittance spectra of the three interlayer films are measured by UV–vis ranging from 300 to 1000 nm. As shown in Figure 1c, the transmittance of the hybrid interlayer is between that of the neat PNDIT‐F3N and PDIN films.

**Figure 2:**
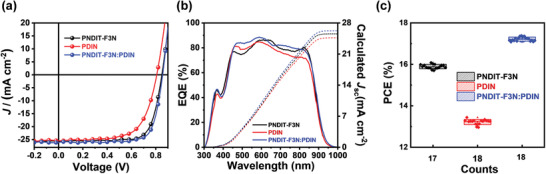
a) *J–V* curves and b) EQE spectra and integrated current density of the best devices. c) PCE distribution of the devices with PNDIT‐F3N, PDIN, and the hybrid interlayer.

To evaluate the cathode interlayer performance, devices with architecture of ITO (indium tin oxide)/PEDOT:PSS/PM6:Y6/interlayer/Ag were fabricated. The current density‐voltage (*J‐V*) are plotted in **Figure** **2**a and the detailed photovoltaic parameters are summarized in **Table** **1**. As demonstrated, the device with PDIN as the cathode interlayer exhibited a *V*_OC_ of 0.80 V, a *J*_SC_ of 25.02 mA cm^–2^, and an FF of 64.31%, resulting in a PCE of 12.9%. When PNDIT‐F3N was used as the interlayer, the devices exhibited a *V*_OC_ of 0.86 V, a *J*_SC_ of 26.06 mA cm^–2^, and an FF of 69.94%, resulting in a PCE of 15.6%. Interestingly, the device with the hybrid interlayer yielded a *V*_OC_ of 0.86 V, an elevated *J*_SC_ of 27.12 mA cm^–2^, and a boosted FF of 74.45%, leading to an outstanding PCE of 17.4%. The difference in PCE is visualized in the statistics chart (Figure 2c), and the optimization of the hybrid interlayer was detailed in Section S8 (Supporting Information). The external quantum efficiency (EQE) spectra of the devices are presented in Figure 2b. The device with hybrid interlayer exhibits a stronger response than that of devices with PNDIT‐F3N and PDIN as the interlayer in the range of 450–620 and 650–810 nm, which agrees with the increased *J*_SC_ and demonstrates improved acceptor contribution. Additionally, calculated *J*_SC_ values of 25.34, 24.68, and 26.23 mA cm^−2^ were obtained by integrating the EQE, in consistent with the *J*_SC_ values measured from the solar simulator (within a 3.5% error, Table 1).

**Table 1:** Photovoltaic parameters of PM6:Y6 OSCs with PDIN, PNDIT‐F3N, and the hybrid interlayer

PNDIT‐F3N:PDIN	*V*_OC_ [V]	FF [%]	*J*_SC_ [mA cm^−2^]	*J*_cal_^a)^ [mA cm^−2^]	PCE_max_/PCE_avg_^b)^ [%]
1:0	0.86 ± 0.01 (0.86)	69.58 ± 0.32 (69.94)	25.85 ± 0.16 (26.06)	25.34	15.7/15.6
0.6:0.4	0.86 ± 0.01 (0.86)	74.15 ± 0.24 (74.45)	26.88 ± 0.21 (27.12)	26.23	17.4/17.1
0:1	0.80 ± 0.01 (0.80)	64.14 ± 0.14 (64.31)	24.86 ± 0.14 (25.02)	24.68	12.9/12.6

^a)^ The *J*_EQE_ calculated from external quantum efficiency (EQE) curve; ^b)^ statistical data obtained from 15 devices.

**Figure 3:**
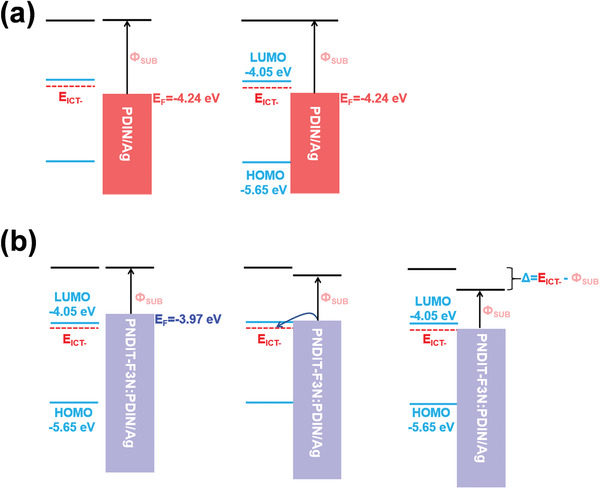
Schemes of the energy‐level alignment before and after contact, when a) PDIN/Ag and b) PNDIT‐F3N:PDIN/Ag were used as the cathode.

Notably, when the cathode interlayer PNDIT‐F3N and the hybrid interlayer were used, all the devices parameters were better than that of the device with PDIN as listed in Table 1. It was reported that the *V*_OC_ was determined by the electrode work function difference based on the metal–insulator–metal model.^[^ ^48^ ^]^ Thus, to understand the increasement in *V*_OC_, the energy level alignment at the active layer/cathode interface were checked. When PDIN/Ag were used as the cathode and got contact with the active layer, the vacuum level aligned, resulting directly pinning of cathode Fermi‐level at the energy level of −4.24 eV (**Figure** **3**a). Since Fermi‐level energy of PNDIT‐F3N/Ag and PNDIT‐F3N:PDIN/Ag were lower than the LUMO level of Y6, the evolution of the organic/cathode contact happened in a same way. While PNDIT‐F3N and the hybrid interlayer were used, the electrons can begin to flow from the electrode to the active layer at the interface until the Fermi‐level was aligned with *E*_ICT‐_ (Figure 3b). At this equilibrium condition, a potential (dipole) at the interface that up‐shifts the vacuum level was created.^[^ ^49^, ^50^ ^]^ As such, no energy was lost by transferring an electron to/from the active layer across the interface. Due to the identical anode and active layer in all devices, the only energetic difference came from the cathode. Hence, the noticeable difference in Fermi‐level pinning caused the incasement in *V*_OC_ when PNDIT‐F3N and the hybrid interlayer were used.

**Figure 4:**
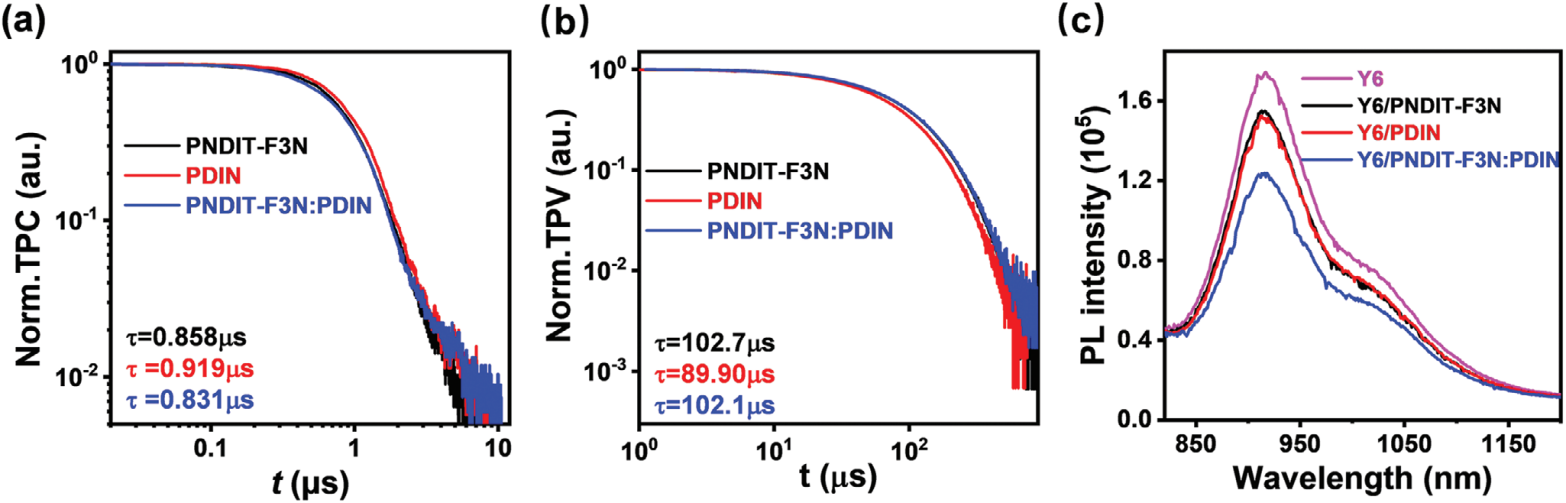
a) Normalized TPC of devices with PNDIT‐F3N, PDIN, and the hybrid interlayer, b) normalized TPV of devices with PNDIT‐F3N, PDIN, and the hybrid interlayer (at open circuit condition w/o background light illumination), and c) photoluminescence spectra of Y6 in neat film and blend films of Y6 with cathode interlayers.

To gain insight on the increasement of FF and *J*_SC_, charge extraction and recombination in the devices were examined. Owing to the identical active layer and anode, the behaviors of charges generated were significantly affected by the cathode interlayer. Transient photocurrent (TPC) measurements can provide information on the charge extraction by fitting the current decay with mono‐exponential decay model. The charge extraction time of the devices with PNDIT‐F3N, PDIN and hybrid interlayer were 0.858, 0.919, and 0.831 µs, (**Figure** **4**a), indicating inefficient charge extraction from devices with PDIN as the cathode interlayer.^[^ ^51^ ^]^ Charge recombination information can be attained from transient photovoltage (TPV) measurements.^[^ ^52^, ^53^, ^54^ ^]^ Usually, the device was excited by a pulse laser and held at open circuit condition with illumination, so the generated charge cannot be extracted. The voltage decay time constant was considered as the charge carrier lifetime. Here, due to the limitation of the experiment, we performed the TPV measurements without illumination. The devices with PNDIT‐F3N and the hybrid interlayer showed longer lifetime, indicating less charge recombination in these devices (Figure 4b). Because the only difference of these devices was the interlayer, trap assisted recombination was ascribed as the dominate recombination route in device with PDIN. Consequently, better charge extraction and less charge recombination led to the improved FF.

**Figure 5:**
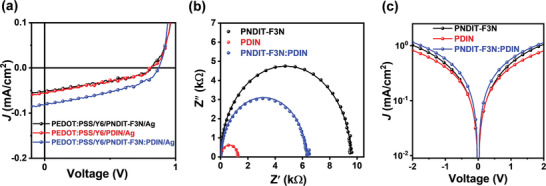
a) *J–V* curves under illumination of devices with Y6 only as the active layer, b) Nyquist plot of three CILs based devices, and c) *J–V* curves in dark of Schottky‐ junction devices based on different cathode interlayers.

On the other hand, we attributed the improved acceptor contribution to the increasement of *J*_SC_ visualized in the EQE spectra. It was pointed out that the performance of device with PM6:Y6 was limited by the contribution of Y6.^[^ ^24^, ^55^ ^]^ Hence, if the acceptor contribution can be improved, the device performance can get better. Except increasing hole transfer efficiency from photoexcited Y6 to PM6, exciton dissociation with the cathode interlayer should be an alternative approach. Therefore, we first measured the photoluminescence (PL) to investigate the exciton behaviors from the acceptor Y6 to the cathode interlayer.^[^ ^56^, ^57^ ^]^ Thin films of neat Y6 and combination of Y6/cathode interlayer was photoexcited with wavelength of 808 nm, and the PL intensity of each film was depicted in Figure 4c. PL quenching was observed in all films. The quenching efficiency of 11.1% (Y6/PNDIT‐F3N), 12.5% (Y6/PDIN), and 29.1% (Y6/hybrid interlayer) was calculated, suggesting that the excitons generated in Y6 can dissociate to the cathode interlayer. To verify the existence of the charge transfer, devices with Y6 as the active layer was fabricated and the *J‐V* curves were recorded (**Figure** **5**a). When the hybrid interlayer was used, the simultaneously improved device parameters indicated that charge transfer happened after the exciton dissociation. As a result, it can be reasonably inferred that the introduction of the hybrid interlayer can substantially improve the exciton dissociation efficiency and enhance the charge transfer, thus enhancing the efficiency.

As a powerful tool to analyze the interfacial properties in devices, impedance spectroscopy (IS) was widely used in OSCs.^[^ ^50^, ^58^, ^60^ ^]^ To attain the Nyquist plots, the devices were measured at frequencies from 1 MHz to 20 Hz under dark condition (Figure 5b). The equivalent circuit was essential to understand the two arcs at low and high frequency regions and provide information on charge transport resistance, recombination resistances, and the chemical capacitances. In this measurement, the equivalent circuit includes two parallel R‐CPE elements connected in series was chose. *R* here represents resistance and CPE is abbreviation of constant phase element, which is a nonideal capacitance related to nonhomogeneities. Additional notes on IS analyze were detailed in Section S5 (Supporting Information) and the final equivalent circuit was presented in Figure S2 (Supporting Information). Due to the similarity of the device structures, the *R*_S_ of the three devices are comparable, but the series resistance of the device with hybrid interlayer is the smallest. The *R*_bulk_ values of the three devices were 76.3, 91.65, and 76.4 Ω, respectively, indicating that the PNDIT‐F3N and hybrid interlayer have similar interfacial transport resistance, which may result from the better conductivity of the hybrid interlayer (details in Figure 5c and Table S2 (Supporting Information)). It was reported that the higher the system recombination resistance, *R*_rec_, the lower the nongerminate recombination rate. Values of *R*_rec_ were 9.41, 1.16, and 6.18 kΩ for the devices with PNDIT‐F3N, PDIN, and the hybrid interlayer, respectively. This result is evidenced that the device with PDIN suffered larger extent of trap‐assisted recombination compared with those of the other devices.

To demonstrate the feasibility of the hybrid cathode interlayer, we fabricated all‐polymer solar cells with the PNDIT‐F3N:PDIN as the cathode interlayer and PM6:PY‐IT as the active layer. The efficiency of the all‐polymer solar cell was improved to from 14.9% to 15.6% listed in Table S4 (Supporting Information). The straightforward yet practical design strategy was further evidenced by the interlayer combination of PNDIT‐F3N:PDINO. When PM6:Y6 was casted as the active layer, maximum PCE of 17.3% was attained with FF of 78.33% presented in Table S5 (Supporting Information). Cathode interlayer plays an important role in OSCs and the polymeric/small molecule hybrid strategy as cathode interlayer proves to be promising approach for enhancement of PCE of OSCs.

## 3 Conclusion

In summary, we reported a hybrid interlayer strategy and obtain an improved PCE based on single junction OSCs with PM6:Y6 as the active layer. Compared to devices with PNDIT‐F3N and PDIN as the cathode interlayer, the device with the hybrid interlayer yielded higher FF of 74.45% and *J*_SC_ of 27.12 mA cm^−2^, resulting in a high PCE of 17.4%. The introduction of the hybrid interlayer led to well aligned energy levels and improved transport resistance due to its lower work function and high conductivity. Furthermore, device with the hybrid interlayer showed enhanced exciton dissociation, and reduced trap‐assistant recombination compared with those of the devices with PNDIT‐F3N and PDIN. The feasible hybrid method was validated by altered electron acceptors and polymeric/small molecule combinations. Our results demonstrated that this hybrid interface engineering strategy is simple and effective way to improve the performance of OSCs, and it is important to revisit and evaluate the reported interlayers by paring them with the emerged FERAs.

## Conflict of Interest

The authors declare no conflict of interest.

## Supporting information

Supporting Information

## Data Availability

The data that support the findings of this study are available from the corresponding author upon reasonable request.
