# Supplementary material for: Hybrid Cathode Interlayer Enables 17.4% Efficiency Binary Organic Solar Cells
Source: Adv Sci (Weinh). 2022 Jan 18;9(8):2105575. doi: 10.1002/advs.202105575 (PMC8922103; doi:10.1002/advs.202105575)
Supplement: Supplementary file 1 — Supporting Information [file ADVS-9-2105575-s001.pdf]

ADVANCED SCIENCE
Open Access

Supporting Information

for *Adv. Sci.*, DOI 10.1002/advs.202105575

Hybrid Cathode Interlayer Enables 17.4% Efficiency Binary Organic Solar Cells

Hang Song, Dingqin Hu, Jie Lv, Shirong Lu, Chen Haiyan* and Zhipeng Kan**

ADVANCED SCIENCE
Open Access

Supporting Information

for *Adv. Sci.*, DOI: 10.1002/advs.202105575

Hybrid cathode interlayer enables 17.4% efficiency binary organic solar cells

Hang Song, Dingqin Hu, Jie Lv, Shirong Lu, Chen Haiyan* and Zhipeng Kan**

Supporting Information

Hybrid cathode interlayer enables 17.4% efficiency binary organic solar cells

Hang Song, Dingqin Hu, Jie Lv, Shirong Lu, Chen Haiyan* and Zhipeng Kan**

H. Song, D. Hu, J. Lv, H. Chen, Prof. S. Lu, Prof. Z. Kan
Chongqing Institute of Green and Intelligent Technology, Chinese Academy of Sciences
Chongqing 400714, China
E-mail: lushirong@cigit.ac.cn; chenhaiyan@cigit.ac.cn; kanzhipeng@cigit.ac.cn
H. Song
College of Materials Science and Engineering, Chongqing University of Technology
Chongqing 400054, China
D. Hu, H. Chen
Chongqing University, 174 Shazhengjie, Shapingba, Chongqing 400044, China
J. Lv, D. Hu, H. Chen, Prof. S. Lu, Prof. Z. Kan
Chongqing School, University of Chinese Academy of Sciences (UCAS Chongqing)
Chongqing 400714, China

1. General Experimental Details

All materials are dissolved in a nitrogen atmosphere and solvents are purified and dried from an appropriate desiccant using standard techniques prior to use. Unless otherwise stated, reagents obtained from commercial sources can be used without further purification.

2.Materials

All materials were supplied by commercial suppliers: ITO (youxuan Technology), PEDOT: PSS (Clevios P VP AI. 4083 (Heraeus)), PM6 (organtec Ltd.), Y6 (Derthon Ltd.), PNDIT-F3N (Sigma-Aldrich Ltd.), PDIN (Derthon Ltd.), chloroform (J&K Sciences Ltd.), isopropanol (J&K Sciences Ltd.) and Ag (ZNXC Ltd.). And all reagents and solvents were used directly, without further purification

3. UV-Vis Spectroscopy

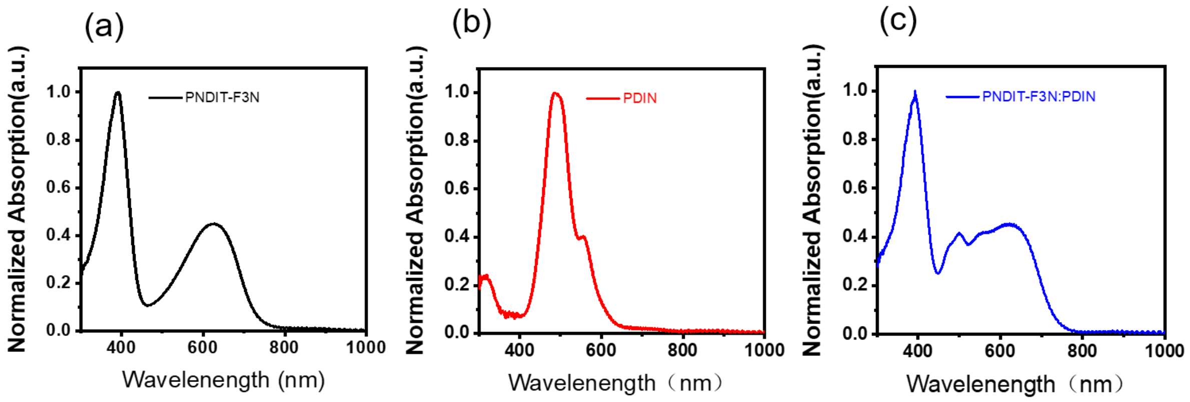

Figure S1. Normalized UV-Vis absorption spectra of ETL films. (a)PNDIT-F3N (b) PDIN (c)PNDIT-F3N:PDIN

4. Details on the measurement of TPC/TPV :

The transient photocurrent (TPC) was tested at short-circuit condition with a 50-ohm resistance in series and transient photovoltage (TPV) measured at open circuit condition with a 1M-ohm resistance in series. The pulse light is a 10 ns laser with wavelength of 532 nm. All measurements were done in dark. The laser average intensity was 230uWcm^{-2}.

5. Impedance Spectroscopy (IS)

IS was conducted using a commercially available Autolab system (Metrohm) electrochemical workstation. The measurements were performed using conditions that had an applied bias of open circuit voltage of OPV in the dark, and a frequency ranging from 1 MHz to 20 Hz.

Table S1. Electrochemical resistance values of the three CILs based devices.

	$R_s(\Omega)$	$R_{bulk}(\Omega)$	$R_{surface}(K\Omega)$
PNDIT-F3N	10.83	76.30	9.14
PDIN	13.91	91.65	1.16
PNDIT-F3N:PDIN	10.42	76.40	6.18

Figure S2. equivalent circuit

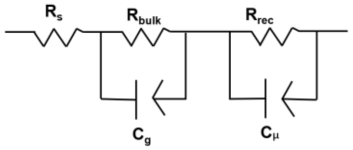

We use impedance spectroscopy (IS) to analyse the simulated interface resistance, IS is a widely used tool in OSC characterization due to its ability to analyze the device charge transfer properties . Impedance (Z'(Ω)) as x-axis and negative imaginary part (-Z''(Ω)) as y-axis. In general, all the Nyquist plots consist of two arcs at low and high frequency regions, performed at frequencies from 1 MHz to 20 Hz, under dark condition.which require two sets of parallel RC elements to fitting carrier transport information. The

equivalent circuit consists of two parallel R-CPE elements connected in series. Here, R represents resistance and CPE (Constant Phase Element) is a non-ideal capacitance, related to non-homogeneities such as porosities and roughness. Rs represents series resistance that accounts the resistance of metallic wires, and ohmic components, such as, ITO and Ag electrodes. Rbulk is the resistance associated with electron transport through the interfacial layers and Cg is the geometric capacitance representing the dielectric component of the diode. The low frequency element of the impedance response is associated with the recombination resistance (Rrec) and chemical ， capacitance (Cμ) of the system. Rrec is associated only with non-geminate recombination because the measurements were performed under dark conditions and no photocarriers were generated. Cμ is known as the distributed chemical capacitance. In OSCs, the chemical capacitance is controlled by the charge carriers injected from the contact. The fitted electronic parameters of the equivalent circuits(Figure S2) are listed in Table S1. As from the fitting results, three CILs based devices with similar Rs because of the same device structure.Where the hybrid interface is the lowest and the battery string is the smallest,this is the one reasons leading to the enhancement of JSC and FF.The R2 values of the PNDIT-F3N, PDIN and hybrid interlayer CILs based devices are 76.3 Ω, 91.65 Ω, 76.4 Ω, respectively. The Rrec for the three was 9.41, 1.16 and 6.18 KΩ respectively, with the larger srec relative to the PDIN unit facilitating a reduction non-geminate recombination.

6. Electrical conductivity

Figure S3 Sandwich structure to measure verticall electron conductivity

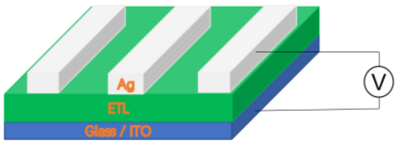

Table S2. Electrical conductivity

	Thickness(nm)	Conductivity(S/cm)
PNDIT-F3N	80±3	5.17E-05

PDIN	45±4	4.15E-05
PNDIT-F3N：PDIN	73±4	5.70E-05

7. Device Fabrication

OSCs were fabricated with a conventional architecture of indium tin oxide (ITO)/poly(3,4-ethylenedioxythiophene): polystyrene sulfonate (PEDOT:PSS)/active-layer/poly[(9,9-bis(3′-((N,N-dimethyl)-N-ethylammonium)propyl)-2,7-fluorene)-alt-5,5′-bis(2,2′-thiophene)-2,6-naphthalene-1,4,5,8-tetracaboxylic-N,N′-di(2-ethylhexyl)imide]dibromide(PNDIT-F3N)/Ag. The ITO-coated glass substrates were sequentially cleaned in detergent, deionized water, acetone and isopropyl alcohol for 15min each at room temperature. A 40-nm-thick PEDOT:PSS layer was first spin-cast on top of the ITO substrates at 5,000 r.p.m. for 30 s and then annealed on a hotplate at 150 °C for 15min under ambient conditions. The blend solutions were prepared by dissolving PM6 and Y6 in chloroform solvent with 1-cn content 0.5%. The total concentration of all active layer solutions was maintained at 17 mg/ml. The active layers were generated by spin-coating the blend solutions (the volume used per round is 12μl) at a spin-coating rate of 3,000 r.p.m. for 30 s on the top of PEDOT:PSS with an optimal thickness of 105 nm, and then were thermally annealed at 100 °C for 10 min in a N2-filled glove box. A thin layer (~5nm) of PNDIT-F3N,PDIN or the mix concentration of 0.5mg/m1 was spin-cast on the top of the active layer at a spin-coating rate of 2,000 r.p.m. for 30 s. Finally, a 100-nm-thick Ag electrode was thermally deposited under vacuum conditions of 2×10−4Pa. Following electrode deposition, samples underwent *J-V* testing.The current density–voltage (*J*–*V*) curves of OSCs were tested in N_2 atmosphere by a Keithley 2400 source meter and an AAA grade solar simulator (SS-X50R, Enli Technology Co., Ltd.) along with AM 1.5 G spectra whose intensity was corrected by a certified standard silicon solar cell at 1000 W/m^2. The J-V curves are measured in the forward direction from 0.2 to 1.2 V. The external quantum efficiency (EQE) was measured by a certified incident photon to electron conversion equipment (QE-R) from Enli Technology Co., Ltd.

8. Additional PV Device Performance Data

Table S3. Photovoltaic performance of PM6:Y6 based solar cells with different PNDIT-F3N:PDIN ratio.

Active Layer	PNDIT-F3N:PDIN	Voc (V)	PCE (%)	FF (%)	J_{SC} (mA/cm^2)
PM6:Y6	1:0	0.86	15.6	69.94	26.06
PM6:Y6	0.9:0.1	0.86	16.2	71.44	26.37
PM6:Y6	0.8:0.2	0.86	16.4	72.45	26.45
PM6:Y6	0.7:0.3	0.86	16.9	73.74	26.73
PM6:Y6	0.6:0.4	0.86	17.4	74.45	27.12
PM6:Y6	0.5:0.5	0.86	16.5	72.17	26.48
PM6:Y6	0:1	0.80	12.9	64.31	25.02

9. Additional PV Device Performance Data (PM6:PY-IT)

To demonstrate the feasibility of the hybrid cathode interlayer, we fabricated all polymer solar cells with the PNDIT-F3N:PDIN as the cathode interlayer and PM6:PY-IT as the active layer. The efficiency of the all-polymer solar cell was improved to from 14.9% to 15.6% listed in Table S4.

Table S4. Photovoltaic performance of OSCs composed of PM6:PY-IT with different PNDIT-F3N:PDIN ratio.

PNDIT-F3N:PDIN	V_{OC} (V)	FF (%)	J_{SC} (mA/cm^2)	[a]PCE (%)
0:1	0.93	70.02	22.71	14.9

0.6:0.4	0.93	72.26	23.24	15.6
1:0	0.88	53.04	22.15	10.4

10. Additional PV Device Performance Data (PNDIT-F3N:PDINO)

Apart from the polymer/small molecule combination of PNDIT-F3N:PDIN, it was evidenced that PNDIT-F3N:PDINO works as well though the best composition was not the same. The PCE of PM6:Y6 improved from 16.4% to 17.3% with the hybrid cathode interlayer.

Table S5. Photovoltaic Parameters of the OSCs composed of PM6:Y6 with different PNDIT-F3N:PDINO ratio.

PNDIT-F3N:PDINO	V_{OC} (V)	J_{SC} (mA/cm^2)	FF (%)	PCE (%)
1:0	0.835	25.22	78.04	16.4
0.7:0.3	0.838	26.47	76.99	17.1
0.9:0.1	0.833	26.42	78.33	17.3
0:1	0.831	25.30	68.58	14.4
